# Does patient satisfaction of general practice change over a decade?

**DOI:** 10.1186/1471-2296-10-13

**Published:** 2009-02-08

**Authors:** James Allan, Peter Schattner, Nigel Stocks, Emmae Ramsay

**Affiliations:** 1 Hills Medical Service, Aldgate, South Australia, Australia; 2 Discipline of General Practice, University of Adelaide, Adelaide, South Australia, Australia; 3 Department of General Practice, Monash University, Melbourne, Victoria, Australia

## Abstract

**Background:**

The Patient Participation Program (PPP) was a patient satisfaction survey endorsed by the Royal Australian College of General Practitioners and designed to assist general practitioners in continuous quality improvement (CQI). The survey was been undertaken by 3500 practices and over a million patients between 1994 and 2003. This study aimed to use pooled patient questionnaire data to investigate changes in satisfaction with primary care over time.

**Methods:**

The results of 10 years of the PPP surveys were analyzed with respect to 10 variables including the year of completion, patient age, gender, practice size, attendance at other doctors, and whether the practice had previously undertaken the survey. Comparisons were made using Logistic Generalized Estimating Equations (LGEE).

**Results:**

There was a very high level of satisfaction with general practice in Australia (99% of respondents). An independent indicator of satisfaction was created by pooling the results of 12 questions. This new indicator had a greater variance than the single overall satisfaction question. Participants were shown to have higher levels of satisfaction if they were male, older, did not attend other practitioners or the practice was small in size. A minimal improvement in satisfaction was detected in this pooled indicator for the second or third survey undertaken by a practice. There was however no statistically significant change in pooled satisfaction with the year of survey.

**Conclusion:**

The very high level of satisfaction made it difficult to demonstrate change. It is likely that this and the presentation of results made it difficult for GPs to use the survey to improve their practices. A more useful survey would be more sensitive to detect negative patient opinions and provide integrated feedback to GPs. At present, there are concerns about the usefulness of the PPP in continuous quality improvement in general practice.

## Background

There is an extensive literature on patient satisfaction with health care but only a few that have been specifically designed and validated for their use in continuous quality improvement (CQI). CQI is a management concept that utilizes repeated cycles of data gathering, analysis, action and reappraisal. It seeks consumer feedback and uses this to generate change and improvement in a service. Examples of such surveys include the General Practice Assessment Questionnaire (GPAQ) used by the National Health Service [1-4], and one designed by the European taskforce (EUROPEP) for comparative evaluation of health quality between different countries in Europe. [5-10]

The Patient Participation Program (PPP) is an Australian survey designed by the Royal Australian College of General Practitioners (RACGP) in 1992 – 93 and which had been in use in general practice until 2003.[11,12] It resulted in over a million patients being surveyed from 3500 general practices over a 10 year period. GPs and practices chose to participate in order to provide points for practitioner's vocational registration and later for the practices accreditation.

There are two versions of the survey that we have named 45Q and 60Q according to the number of questions they contain. Each survey encompassed a range of topics including interaction with the doctor, accessibility of care and the range of services available within the practice. The survey was completed by the patient in the waiting room before and after a consultation. The initial 45Q survey was validated by factor analysis.[11,12] In 1999 the instrument was modified to include additional questions designed for practice accreditation.

In the literature the longest time period over which patient satisfaction has been analysed in general practice is only 15 months and the study involved showed that patient satisfaction improved over time. [13] No articles on the measurement of patient satisfaction over a ten year period were discovered in a thorough review of the literature. Unfortunately, the few longitudinal satisfaction studies that do exist, such as those originating from health funds in the United States, have had significant methodological limitations. [14]

The aim of this study was to investigate whether patient satisfaction varied with practice characteristics and time. It was postulated that changes in patient satisfaction might, in part, reflect consumer/patient acceptance of broader changes in general practice.

The secondary aim was to determine whether undertaking the PPP program would improve subsequent patient satisfaction results from participating practices. This would be a reasonable assumption if the practices were undertaking CQI processes effectively.

## Methods

The survey results were stored by the RACGP in numerous ASCII databases. (ASCII is a standard 7-bit code for the transmission of data). The RACGP gave permission to undertake secondary data analyses, provided anonymity was maintained. The data were converted into two excel spreadsheets and analysed using Logistic Generalized Estimating Equations (LGEE).

### Development of indicators of satisfaction

**Figure 1:**
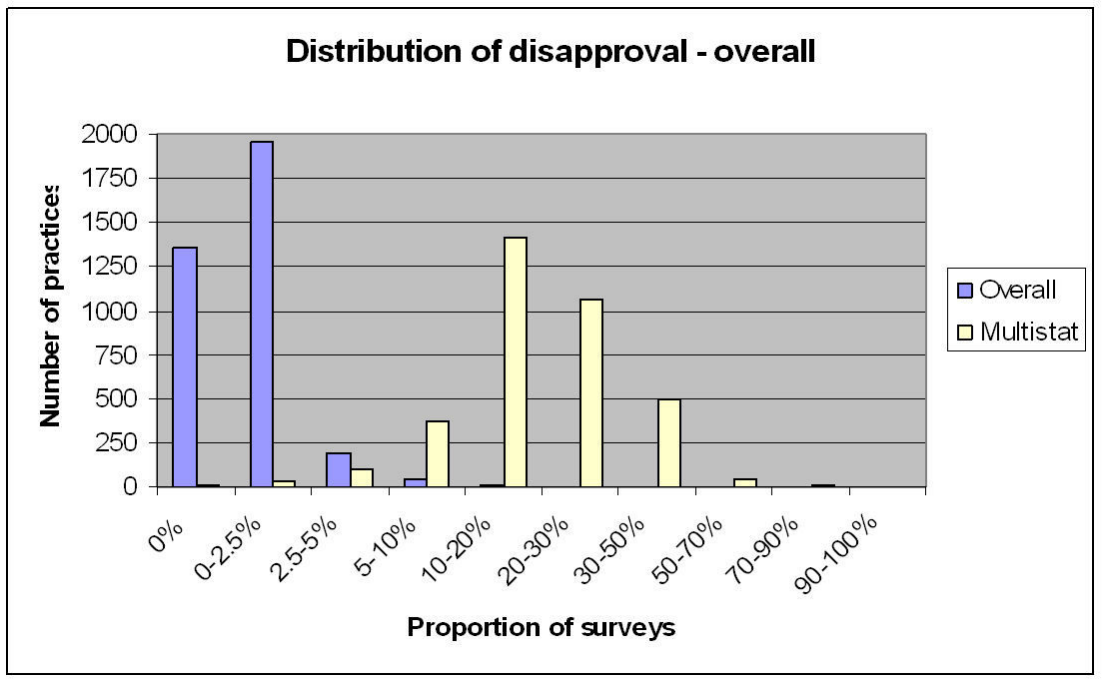
**note the categories on the x-axis are not of equal size**.

Each of the two surveys contained a single question that enquired about the respondent's overall level of satisfaction with the practice. (This variable we named "overall") The four point answer scale was dichotomized into satisfied ('very satisfied' and 'satisfied') and unsatisfied responses('dissatisfied' and 'very dissatisfied'). Despite excellent face validity, this question had problems as an indicator of satisfaction in that there was very poor response variability (figure 1). More than 99% of respondents were fully satisfied with their practice.

A separate indicator or measure of satisfaction was derived, in the absence of any such indicator in the original survey. We chose 12 questions that represented a range of important determinants of satisfaction and had almost identical wording in the two versions of the questionnaire. (45Q and 60Q) Refer to table 1. We have named this indicator "multistat" (pooled results of multiple statistics). This indicator was dichotomized into a group who were satisfied with all 12 items and a group that were dissatisfied with one or more items. A small pilot of 28 patients completing both 45Q and 60Q surveys concurrently indicated 82% concordance of the "multistat" indicator derived from each version of the survey.

**Table 1:** Comparison of wording for overall satisfaction question and the 12 selected questions from the 45-Q and 60-Q survey

PPP 45 Question survey		PPP 60 Question survey	
16	In general how satisfied are you with the medical care you receive at this practice?	55	Overall how satisfied were you with the consultation?
PPP 45 Question survey		PPP 60 Question survey	
20	Are you satisfied with the ease of making an appointment to see the doctor	35	Were you able to obtain your appointment at a time that was convenient for you?
23	Are you satisfied with the ease of seeing the doctor out of normal working hours	30	How easy have you found it to see a doctor out of hours?
24	Are you satisfied with the ease of having the doctor see you at home	29	How easy have you found it to obtain a home visit during office hours?
26	Are you satisfied with the amount of time the doctor spends with you	38	Did the doctor spend enough time with you?
28	Are you satisfied with the handling of accounts by the doctors office	21	Are you satisfied with the handling of accounts by the doctors office?
29	Are you satisfied with the doctors ability to deal with children	22	Are you satisfied with the doctor's ability to deal with children?
30	Are you satisfied with the doctor's willingness to spend time with you	23	Are you satisfied with the doctor's willingness to spend time with you?
31	Are you satisfied with the doctor's willingness to answer your questions	24	Are you satisfied with the doctor's willingness to answer your questions
32	Are you satisfied with the respect shown to you by the doctor	17	Are you satisfied with the respect shown to you by your doctor?
34	Are you satisfied with the doctors ability to treat your problems	25	Are you satisfied with the doctors ability to treat your problems?
35	Are you satisfied with the doctors concern about you problems	26	Are you satisfied with the doctor's concern about your problems?
37	Are you satisfied with the ability to choose which doctor you see	10	Are you able to see the doctor of your choice at this practice?

(Abreviations: sat. – satisfied with, WR – waiting room, Dr – doctor, SE – side effects, Rx – medication, avail. – availability)

### Analysis

Multivariable analysis was undertaken comparing patient satisfaction, as measured by the "overall" and "multistat" indicators, to 10 independent variables. The independent variables were the patient's age, gender, years attending the practice and whether they saw a doctor from another practice, the practice size, the practice location, using the Accessibilty and Remoteness Index of Australia (ARIA code)[15], and socioeconomic status, using the Socio Economic Indexes For Areas (SEIFA code)[16], the year the survey was conducted, the number of times a practice had conducted the survey and the questionnaire that had been used. Logistic Generalized Estimating Equations (LGEE)[17,18] was chosen for the analysis. LGEE analyses data in discrete clusters, regarding all of the surveys from a single practice as being separate from surveys from other practices.

## Results

**Figure 2:**
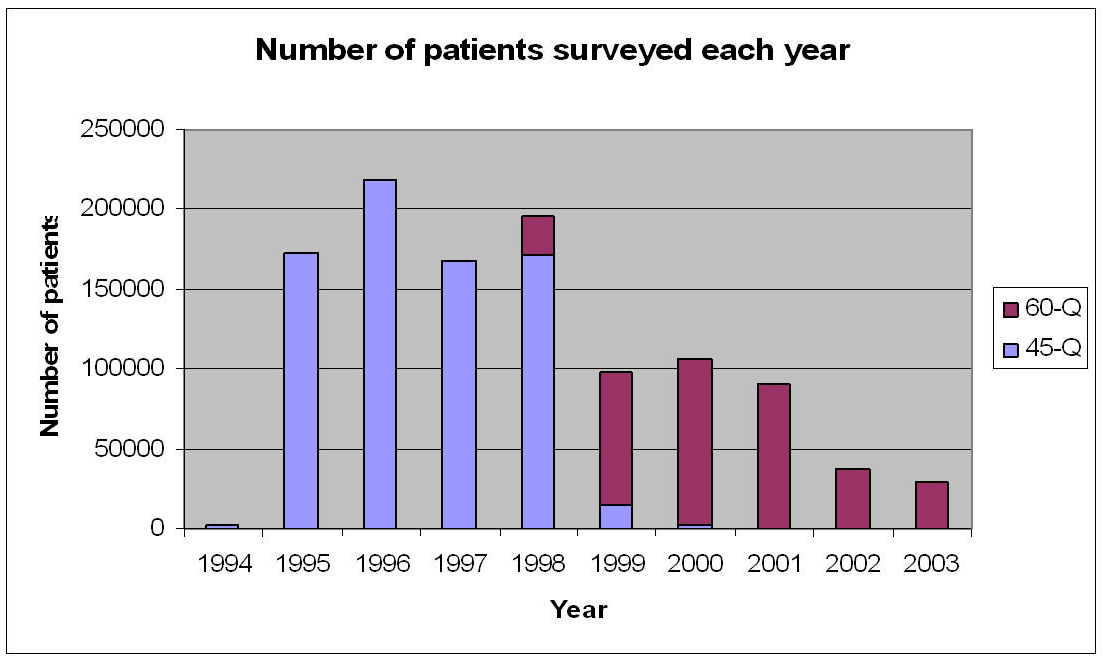
**Patients surveyed by year**.

The completed database included surveys collected from 1,119,688 patients. This represented 10,709 survey episodes undertaken by 3,554 distinct practices. We have no information on response rates. Also it was not possible to match 845 surveys (7.9%) to a known practice and these results were excluded from analysis. The earliest survey was scanned on the 12^th ^January 1994 and the latest on the 8^th ^December 2003. After a peak of 218,033 in 1996, the number of surveys per year has dropped to 28,448 in 2003. Figure 2 gives a breakdown of the number of patients surveyed each year.

Figure 1 illustrates the distribution of practices according to the proportion of dissatisfied responses for each of the two indicators (overall satisfaction and multistat) within discrete practices.

The median dissatisfaction rate for the "overall" indicator was only 0.5% with an intraquartile range from 0%–1.2%. The median dissatisfaction rate for the "multistat" indicator was more substantial at around 18% with a intraquartile range from 12%–26%.

It was noteworthy that 2 practices stood out with over 20% of patients dissatisfied (overall), and 2 practices had over 90% of patients dissatisfied with at least one of the 12 selected items (multistat).

Within both surveys, the questions with the most dissatisfaction included appointment availability, access to home visits, access to after hour care, waiting time, discussion of the costs of treatments and the cost of investigations.

### Analysis

**Figure 3:**
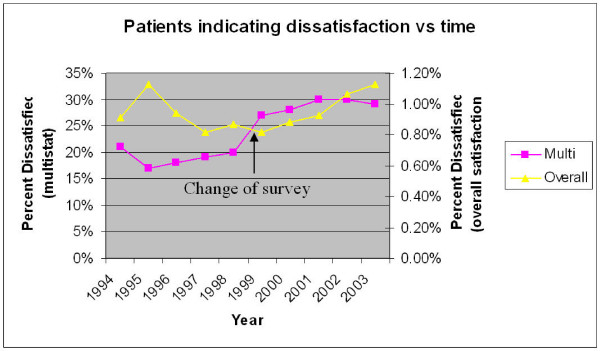
**note the 'overall' and 'multistat' have different y axes**.

Figure 3 demonstrates the level of dissatisfaction in each of the two indicators for each year of the survey. The apparent change in the multistat indicator in 1999 is presumably due to the switch from the 45Q to the 60Q survey.

Multivariable analysis showed that satisfaction was related to all the variables examined except year of survey for the multistat (i.e. time). Although the overall indicator demonstrated significant change with time(p = 0.01) the size of this change was very small (Table 2) rendering this result unimportant or without meaning.

**Table 2:** Multivariable analysis of 10 independent variables in relation to the two satisfaction variables.

Independent Variable	Overall	Multistat
		
	p	p
Patient Age	<.01	<.01
Patient Gender	<.01	<.01
Yrs patient has attended Practice	0.02	<.01
Pt attends different doctor	<.01	<.01
ARIA code of practice	<.01	<.01
SEIFA code of practice	<.01	<.01
Number of GPs in practice	<.01	<.01
Sequence (1^st^, 2^nd ^survey etc)	0.82	<.01
Year of survey	0.01	0.27
Questionnaire	<.01	<.01

The odds ratios (OR) for the independant variables in the multivariable analysis are listed in tables 3 and 4. An OR greater than 1 indicates higher dissatisfaction. This was shown to diminish as measured by the "multistat" indicator with advancing patient age, male gender, smaller practice size, patients who do not visit other doctors, attendance at practices in highly accessible areas (ARIA) and high SE areas (SEIFA). Dissatisfaction slightly increased with respect to duration of attending a practice, particularly after the first year. All of these changes were statistically significant. The Overall indicator gave similar results with the exception of gender, survey instrument, and years attending the practice, where satisfaction was influenced in the opposite direction.

**Table 3:** Multivariable analysis – Odds Ratios for Dissatisfaction "Overall"

**Variable**	**Category**	**Estimate**	**SE**	**Odds Ratio**
Age	14 yrs or less	0.169	(0.194)	1.184
	15–24 yrs	0.265	(0.093)	1.303
	25–44 yrs	0.172	(0.081)	1.188
	45–64 yrs	0.053	(0.081)	1.054
	65–74	-0.130	(0.089)	0.878
	75+	-	-	-
Gender	Female	-0.116	(0.043)	0.890
	Male	-	-	-
Years Attending Practice	0–1 yrs	0.179	(0.058)	1.196
	2–3 yrs	0.108	(0.055)	1.114
	4–5 yrs	0.096	(0.051)	1.101
	6+ years	-	-	-
See Another Doctor	No	-0.922	(0.045)	0.398
	Yes	-	-	-
ARIA	Highly Accessible	-1.268	(0.356)	0.281
	Accessible	-0.980	(0.359)	0.375
	Moderately Accessible	-0.899	(0.366)	0.407
	Remote	-1.123	(0.394)	0.325
	Very Remote	-	-	-
Sequence	1	-0.300	(0.302)	0.741
	2	-0.297	(0.292)	0.743
	3	-0.290	(0.293)	0.748
	4	-0.124	(0.312)	0.883
	5	0.019	(0.467)	1.019
	6	-	-	-
Year	1994	-0.403	(0.413)	0.668
	1995	-0.325	(0.285)	0.723
	1996	-0.542	(0.281)	0.582
	1997	-0.648	(0.287)	0.523
	1998	-0.623	(0.289)	0.536
	1999	-0.452	(0.293)	0.636
	2000	-0.337	(0.209)	0.714
	2001	-0.347	(0.286)	0.707
	2002	-0.346	(0.345)	0.708
	2003	-	-	-
Survey Version	45	0.397	(0.153)	1.487
	60	-	-	-
Practice Size		0.081	(0.008)	1.084
SEIFA	Low	0.282	(0.066)	1.326
	Low/Medium	0.285	(0.109)	1.330
	Medium/High	0.154	(0.098)	1.166
	High	-	-	-
Intercept		-2.538	(0.494)	

**Table 4:** Multivariable analysis – Odds Ratios for Dissatisfaction – Multistat

**Variable**	**Category**	**Estimate**	**SE**	**Odds Ratio**
Age	14 yrs or less	0.638	(0.052)	1.893
	15–24 yrs	0.679	(0.025)	1.972
	25–44 yrs	0.847	(0.023)	2.333
	45–64 yrs	0.540	(0.021)	1.716
	65–74	0.120	(0.021)	1.127
	75+	-	-	-
Gender	Female	0.118	(0.010)	1.125
	Male	-	-	-
Years Attending Practice	0–1 yrs	-0.494	(0.018)	0.610
	2–3 yrs	-0.156	(0.016)	0.856
	4–5 yrs	-0.045	(0.015)	0.956
	6+ years	-	-	-
See Another Doctor	No	-0.489	(0.015)	0.613
	Yes	-	-	-
ARIA	Highly Accessible	-0.405	(0.166)	0.667
	Accessible	-0.271	(0.168)	0.763
	Moderately Accessible	-0.270	(0.175)	0.763
	Remote	-0.385	(0.206)	0.680
	Very Remote	-	-	-
Sequence	1	-0.190	(0.157)	0.827
	2	-0.257	(0.154)	0.773
	3	-0.229	(0.154)	0.795
	4	-0.203	(0.156)	0.816
	5	-0.195	(0.148)	0.823
	6	-	-	-
Year	1994	-0.119	(0.169)	0.888
	1995	-0.152	(0.115)	0.859
	1996	-0.095	(0.110)	0.909
	1997	-0.069	(0.106)	0.933
	1998	-0.056	(0.105)	0.946
	1999	0.006	(0.096)	1.006
	2000	-0.011	(0.095)	0.989
	2001	0.068	(0.098)	1.070
	2002	-0.035	(0.108)	0.966
	2003	-	-	-
Survey Version	45	-0.515	(0.051)	0.598
	60	-	-	-
Practice Size		0.061	(0.005)	1.063
SEIFA	Low	0.220	(0.032)	1.246
	Low/Medium	0.165	(0.410)	1.179
	Medium/High	0.094	(0.046)	1.099
	High	-	-	-
Intercept		-0.500	(0.239)	

**Figure 4:**
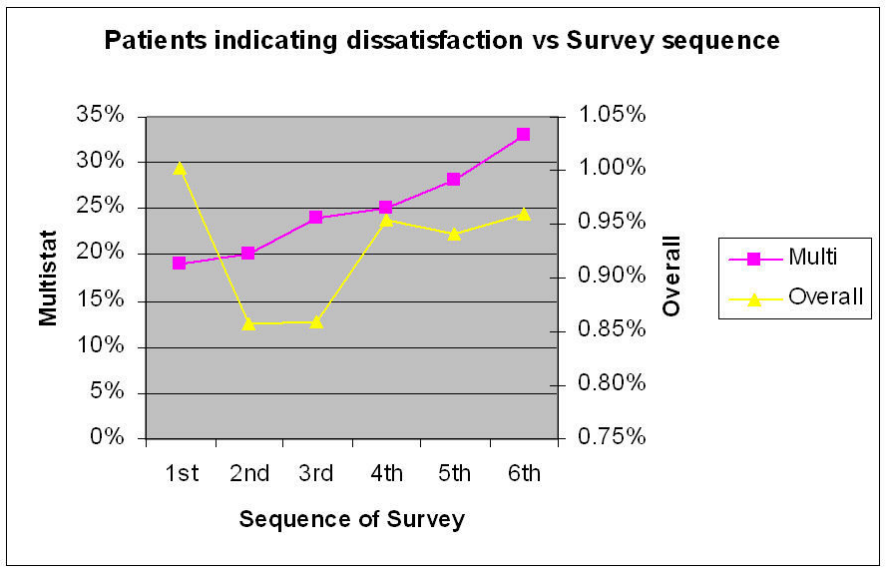
**Sequence indicates the order in which a survey was conducted, ie first survey, second, third etc**. note the number of practices participating drops off as the sequence increases.

Figure 4 compares satisfaction according to survey sequence (the first, second, third or more survey conducted by a given practice). This should not be confused with the year of the survey. The change in relation to the Overall indicator did not reach significance. Although dissatisfaction with the multistat appeared to increase with each subsequent survey episode, the multivariable analysis indicates otherwise. The odds ratio (table 4) show that there was a small drop in dissatisfaction with the second and third surveys and then 4^th ^and 5th surveys show the same level of dissatisfaction as the initial survey. It is noteworthy that the relative magnitude of this decrease in dissatisfaction (multistat) between first and second surveys is only 7%. (Odds ratio = 1.07) In other words dissatisfaction has only dropped from around 21% to 19.5%.

## Discussion

The primary aim of this analysis was to look at change in patient satisfaction over time. On multivariable analysis we found that there was a significant change in the "overall" indicator but not in the more robust "multistat". The actual size of the change in "overall" satisfaction was less than 1 percent and must be regarded as inconsequential. Accordingly we conclude there was no meaningful change with time.

We had analyzed over a million surveys and it is unlikely that the survey lacks power.

It could be argued that the survey lacked sensitivity. The survey however performs as well as other surveys. Female patients, younger patients and those who regularly attend other doctors exhibited more dissatisfied responses. (multistat) Also, there was greater dissatisfaction with larger sized practices. These findings have been independently found in other surveys. [19-26] The replication of these finding offers a degree of criterion validity to the survey instruments.

It should be noted that the two indicators demonstrated opposite effects on some variables (gender, survey instrument, years attending the practice), suggesting that they have slightly different meanings. We have found the multistat to be more useful as it has greater response variability.

Alternatively the effect with time could have been confounded by the change in survey instrument midway through the 10 year period. We attempted to ameliorate this by including it as a variable in the multivariable analysis.

Despite the survey being a sincere attempt to provide practices with opportunities to practice CQI, there may have been limitations in the administration of the instrument. It is not possible to control the selection of patients, or the introduction of bias from reception staff or practitioners. Regardless, it represents a real life attempt at providing a survey for a large number of practices over a prolonged period of time.

The fact that patient satisfaction did not change in a decade that saw major changes to the structure of general practice in Australia, like accreditation, Divisions of General Practice, changes in the GP demographic, vocational registration and continuing medical education is itself surprising. It could be that patients remain satisfied despite these changes. It would seem more likely that "patient satisfaction" in the PPP survey did not measure satisfaction with the structure of general practice, either on the micro or macro scale. It implies that patient satisfaction may in fact be relatively stable over time.

The secondary aim was to compare satisfaction from practices undertaking the PPP for the first time, with second, third and subsequent surveys. Practices undertaking the program were required to review the results and identifying changes that could be made to their practice. It was hypothesized that subsequent surveys should show improvement in patient satisfaction. Multivariable analysis indicated that only the more robust "multistat" indicator showed significant change. This change however was rather meager. It should be noted that the power of the analysis drops off with the sequence, as fewer practices undertook the larger number of surveys.

This small improvement in patient's perceptions was noted only for practices undertaking the program at the 2nd and 3rd visit. The odds ratio 1.07 (between first and second surveys) represents only a small change from 21% to 19.5% dissatisfaction. The size of this change is so small in magnitude as to be rendered almost meaningless. If there is an improvement in patient satisfaction, it is eroded by the third cycle and is completely lost by the fourth or fifth cycle. In light of this result the effectiveness of the PPP survey as an instrument for CQI should be regarded as questionable.  

The study uncovered several deficiencies in the survey design. These included the lack of an integrated index like the "multistat" in feedback to GPs, and the very high level of satisfaction, leaving no room to register improvement. Although many patient surveys report high satisfaction levels, they often fail to uncover the negative opinions of respondents. [27] In addition it has been noted that GPs are not disposed to respond to negative information.[13,28-30] There was evidence of this effect when we reviewed GP responses to their survey results.

## Conclusion

In conclusion, the PPP has failed to identify changes in patient satisfaction with time, and has shown only small non sustained improvement with subsequent cycles of the program. Although minimal initial improvement in satisfaction was demonstrated, the small magnitude and transience brings it's usefulness in CQI open to question. It could be enhanced if future surveys address some of the major deficiencies of this survey, namely failing to elicit negative feedback from patients, lack of an integrated index and failing to address GP attitudes to negative feedback.

## Abbreviations

45Q: 45 question survey; 60Q: 60 question survey; ARIA: Accessibilty and Remoteness Index of Australia; ASCII: American Standard Code for Information Interchange; CQI: continuous quality improvement; EUROPEP: General Practice Questionaire designed by European Taskforce; GP: General Practitoner; GPAQ: General Practice Assessment Questionnaire; LGEE: Logistic Generalized Estimating Equations; Multistat: Patient satisfaction measure derived from multiple questionnaire statistics; OR: Odds Ratio; p: Probability; PPP: Patient Participation Program; RACGP: Royal Australian College of General Practitioners; SEIFA: Socio Economic Indexes For Areas

## Competing interests

The authors declare that they have no competing interests.

## Authors' contributions

JA conceived and supervised the project. This was undertaken as a Masters thesis by distance education from Monash University, with support from the University of Adelaide Discipline of General Practice and funding supplied by the Primary Health Care Research Evaluation and Development (PHC RED) Program. He negotiated with the Royal Australian College of general Practitioners (RACGP) for access to the data, converted the data into an access database, proposed the research questions and drafted the manuscript. PS is Clinical associate Professor of the department of general practice, Monash University. He supervised the Masters thesis reviewing the proposal, analysis and manuscript. NS is Professor of the Discipline of General Practice at the University of Adelaide and Director of the Primary Health Care Research Evaluation and Development Program (PHC RED Program) at the University of Adelaide. He co-supervised the thesis. ER is a statistician within the discipline of General Practice, University of Adelaide. She undertook the statistical modeling and reviewed the manuscript/analysis. All authors read and approved the final manuscript.

## Pre-publication history

The pre-publication history for this paper can be accessed here:


